# BSND and ATP6V1G3: Novel Immunohistochemical Markers for Chromophobe Renal Cell Carcinoma

**DOI:** 10.1097/MD.0000000000000989

**Published:** 2015-06-19

**Authors:** Kazuya Shinmura, Hisaki Igarashi, Hisami Kato, Kenji Koda, Hiroshi Ogawa, Seishiro Takahashi, Yoshiro Otsuki, Tatsuaki Yoneda, Yuichi Kawanishi, Kazuhito Funai, Tatsuya Takayama, Seiichiro Ozono, Haruhiko Sugimura

**Affiliations:** From the Department of Tumor Pathology (KS, HI, HK, HS); ResearchEquipment Center (YK); Department of Surgery 1 (KF); Department ofUrology(TT, SO), Hamamatsu University School of Medicine, Hamamatsu; Department of Pathology, Fujieda Municipal General Hospital,Fujieda (KK); Division of Pathology, Seirei Mikatahara General Hospital, Hamamatsu (HO, ST); and Department of Pathology (YO); Department of Urology(TY),Seirei Hamamatsu General Hospital, Hamamatsu, Japan.

## Abstract

Differentiating between chromophobe renal cell carcinoma (RCC) and other RCC subtypes can be problematic using routine light microscopy. This study aimed to identify novel immunohistochemical markers useful for a differential diagnosis between chromophobe RCC and other RCC subtypes.

We selected 3 genes (including *BSND* and *ATP6V1G3*) that showed specific transcriptional expression in chromophobe RCC using expression data (n = 783) from The Cancer Genome Atlas (TCGA) database. A subsequent immunohistochemical examination of 186 RCCs obtained in our patient series resulted in a strong diffuse positivity of BSND and ATP6V1G3 proteins (both of which are involved in the regulation of membrane transport) in all the chromophobe RCC specimens (23/23 cases, 100%) but not in the clear cell RCC specimens (0/153 cases, 0%) or the papillary RCC specimens (0/10 cases, 0%). BSND and ATP6V1G3 protein expressions were also detected in renal oncocytoma (13/14 cases, 92.9%) and in the distal nephron, including the collecting duct, in the normal kidney. A computational analysis of TCGA data suggested that DNA methylation was involved in the differential expression pattern of both genes among RCC subtypes. Finally, an immunohistochemical analysis showed lung carcinomas were negative (0/85 cases, 0%) for the expression of both proteins.

These results suggest that BSND and ATP6V1G3 are excellent novel immunohistochemical markers for differentiating between chromophobe RCC and other subtypes of RCC, including clear cell and papillary RCCs.

## INTRODUCTION

Chromophobe renal cell carcinoma (RCC) is a subtype of RCC and constitutes approximately 5% of renal neoplasms.^1^ Patients with chromophobe RCC have a better prognosis than those with conventional clear cell RCC, and more than 90% of patients with chromophobe RCC are alive 5 years after surgery;^2–4^ however, aggressive features and distant metastasis of chromophobe RCC can occur.^5^ Hoffmann et al^6^ previously examined sites of distant metastases in chromophobe RCC patients with initial M0 disease who had experienced metastasis during their follow-up period and found that the lung is the most frequent site of distant metastasis but that metastases of chromophobe RCC can also be found in other tissues.

Histologically, there are 2 major variant types, referred to as the classic (alternatively, typical) variant and the eosinophilic variant in the chromophobe RCC.^7,8^ Although representative cases of chromophobe RCC can be easily distinguished from other renal epithelial tumors based on morphologic features, difficulties in making a proper diagnosis are sometimes encountered in histologically borderline cases; for example, a classic variant of chromophobe RCC might be confused with clear cell RCC, and its eosinophilic variant might be confused with clear cell RCC, papillary RCC, or renal oncocytoma. Therefore, immunohistochemistry is often used to assist in the differential diagnosis of chromophobe RCC in such cases.^7,9^ So far, the frequencies of immunoreactivity for CK7, CD117 (C-Kit), parvalbumin, CDH1 (E-Cadherin), CLDN7 (Claudin 7), CA2 (Carbonic Anhydrase II), EMA, CD82 (KAI1), EPCAM (Epithelial Cell Adhesion Molecule), and CDH16 (KSP-Cadherin) have been reported to be higher in chromophobe RCC than in clear cell RCC and/or papillary RCC,^10–21^ and some antibodies against these antigens are used in practical pathological diagnosis. However, since a subset of chromophobe RCCs that are immunohistochemically negative for these markers and a subset of clear cell RCCs and papillary RCCs that are positive for these markers have also been reported,^10–21^ the results of immunohistochemical analyses are not always reliable.

Microarray analysis has been used as an analytical method for measuring the level of mRNA expression for more than a decade, despite some limitations such as the occurrence of hybridization artifacts and a low dynamic range.^22,23^ Recently, however, the RNA-sequencing (RNA-seq) method has been developed, enabling the aforementioned limitations of microarray analysis to be overcome. Moreover, this method also has an advantage over microarray analysis in terms of some issues, such as the detection of low abundance transcripts, the differentiation of isoforms, and its dynamic range.^24^ Thus, the use of the RNA-seq method might lead to the identification of novel cancer-related changes in mRNA expression. Therefore, to identify novel reliable immunohistochemical markers that are useful for distinguishing between chromophobe RCC and other subtypes of RCC, such as clear cell and papillary RCCs, we globally compared the processed RNA-seq expression data from 66 cases of chromophobe RCC, 519 cases of clear cell RCC, and 198 cases of papillary RCC that were included in The Cancer Genome Atlas (TCGA)^25^ and found 3 candidate genes including *BSND* and *ATP6V1G3*. Next, we examined the expression statuses of these genes in 200 primary renal tumors and 85 primary lung carcinomas, and found that BSND and ATP6V1G3 were highly sensitive and specific markers of chromophobe RCC. Our study suggests that evaluating the expression levels of BSND and ATP6V1G3 could be of great value for distinguishing between chromophobe RCC and other subtypes of RCC.

## MATERIALS AND METHODS

### Collection of Publicly Available TCGA Data

Gene expression data and DNA methylation data for a total of 783 RCC cases, composed of 66 chromophobe RCC cases, 519 clear cell RCC cases, and 198 papillary RCC cases (TCGA public data available in April 2014), were collected from the TCGA data portal (https://tcga-data.nci.nih.gov/tcga). Gene expression data for bladder urothelial carcinoma, breast invasive carcinoma, cervical squamous cell carcinoma and endocervical adenocarcinoma, colon adenocarcinoma, head and neck squamous cell carcinoma, liver hepatocellular carcinoma, prostate adenocarcinoma, rectal adenocarcinoma, stomach adenocarcinoma, and thyroid carcinoma were also collected from the TCGA data portal. The expression data were obtained as processed RNA-seq data in the form of RNA-seq by Expectation Maximization or in the form of Reads Per Kilobase of exon Model per million mapped reads.^26,27^ The RNA-seq by Expectation Maximization or Reads Per Kilobase of exon Model per million mapped reads expression value for each gene was divided by that of the *TBP* gene, which is a control housekeeping gene,^28^ to compare the expression levels. DNA methylation data obtained using the Infinium HumanMethylation450 platform (Illumina, Inc, CA) were shown as the β value.^25^

### Preparation of Tissue Microarray (TMA) Blocks

Paraffin-embedded tissue samples from a total of 200 primary renal tumor cases, composed of 23 chromophobe RCC cases, 153 clear cell RCC cases, 10 papillary RCC cases, and 14 oncocytoma cases, that had undergone surgery at Hamamatsu University Hospital (Japan), Fujieda Municipal General Hospital (Japan), Seirei Mikatahara General Hospital (Japan), or Seirei Hamamatsu General Hospital (Japan) were collected, and 190 cases of them were used for the TMA block. In addition, paraffin-embedded tissue samples from a total of 85 primary lung carcinoma cases, composed of 44 cases of squamous cell carcinoma of the lung and 41 cases of adenocarcinoma of the lung, which had undergone surgery at Hamamatsu University Hospital (Japan) were used for the TMA block. To explain in further detail, the block was prepared by transferring a cylinder of 3-mm diameter from each of the paraffin-embedded tissue samples using a microarrayer (KIN-1; Azumaya, Tokyo, Japan), as previously described.^29^ All the cases used for the immunohistochemical staining were listed in Table 1. This study was conducted with the approval of the Institutional Review Board (IRB) of Hamamatsu University School of Medicine.

**TABLE 1:**
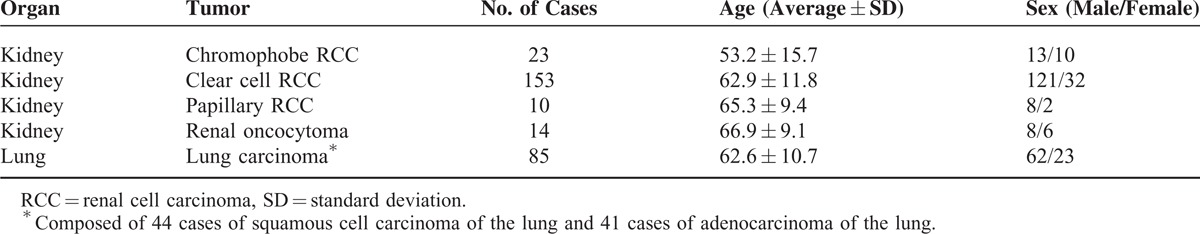
Primary Tumor Cases Used for the Immunohistochemical Analysis in This Study

### Immunohistochemical Staining

Sections of paraffin blocks were used for immunohistochemical staining with an automatic immunohistochemical stainer, the HISTOSTAINER (Nichirei Bioscience, Tokyo, Japan). Briefly, the sections were deparaffinized, rehydrated, and boiled at 96°C for 40 minutes in TE solution (pH 9.0) for antigen retrieval. Endogenous peroxidase activity was blocked by incubation for 5 minutes in a 3% hydrogen peroxide solution. Next, the sections were incubated with a rabbit anti-BSND polyclonal antibody (1:1000; Sigma–Aldrich, St. Louis, MO), a rabbit anti-ATP6V1G3 polyclonal antibody (1:2000; Sigma–Aldrich), or a rabbit anti-FBN3 polyclonal antibody (1:100; Sigma–Aldrich) for 30 minutes at room temperature. After washing, the sections were incubated for 30 minutes at room temperature with an amino acid polymer conjugated with goat antirabbit IgG and horseradish peroxidase (Histofine Simple Stain MAX-PO Kit; Nichirei, Tokyo, Japan). The antigen–antibody complex was visualized with 3,3′-diaminobenzidine tetrahydrochloride, and the sections were counterstained with hematoxylin. The staining intensity for BSND and ATP6V1G3 were graded for each specimen as follows: negative, weakly positive, or strongly positive. Additionally, proportion of positive cells for each specimen in the immunostaining was grouped into 3 categories as follows: none (<1%), partial (1%–90%), and diffuse (≥90%).

### Statistical Analysis

The statistical analysis was performed using a Kruskal-Wallis test or the Spearman rank correlation test. JMP version 9.0 software (SAS Institute, Cary, NC) was used for the analyses.

## RESULTS

**FIGURE 1:**
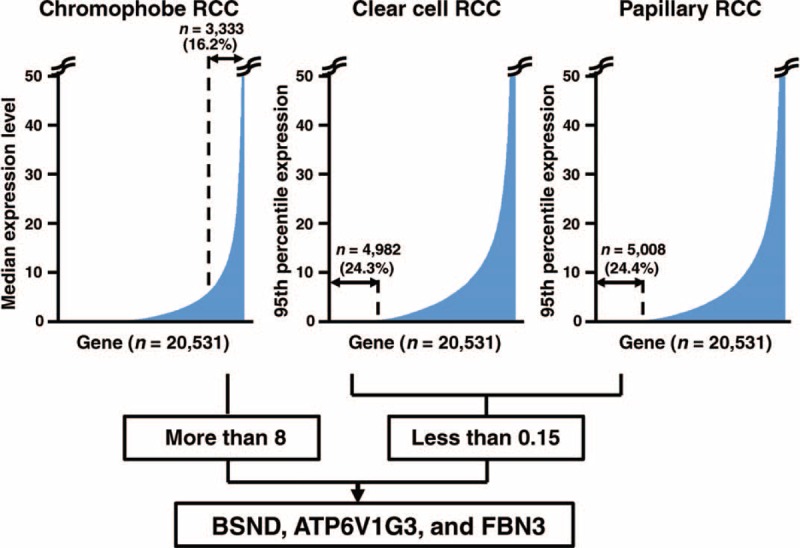
Schematic overview of the algorithm used to identify genes specifically expressed in chromophobe RCC, compared with those expressed in clear cell RCC and papillary RCC, using data from the TCGA database. The median mRNA expression values of all the genes (n = 20,531) in chromophobe RCC (n = 66) were graphed, and the number and percentage of genes whose values were more than 8 are shown in the left panel. The 95th percentile expression values of all the genes in clear cell RCC (n = 519) and papillary RCC (n = 198) were graphed, and the number and percentage of genes whose values were less than 0.15 are shown in the middle panel and right panel, respectively. The expression levels are shown as the RSEM value of each gene, divided by that of the TBP gene. Genes satisfying the following 2 conditions were selected: a median expression value of more than 8 in the chromophobe RCC specimens, and a 95th percentile expression value of less than 0.15 in the clear cell RCC and papillary RCC specimens. RCC = renal cell carcinoma, RSEM = RNA-seq by Expectation Maximization.

To identify immunohistochemistry markers for differentiating between chromophobe RCC and other RCC subtypes, such as clear cell RCC and papillary RCC, we first attempted to compare mRNA expression data, which was based on RNA-seq experiments and was derived from the TCGA database, for chromophobe RCC (n = 66), clear cell RCC (n = 519), and papillary RCC (n = 198). To identify chromophobe RCC-specific genes from whole genes using this data, we selected genes that satisfied the following 2 conditions: (1) a median expression value of more than 8 in the chromophobe RCC specimens, and (2) a 95th percentile expression value of less than 0.15 in the clear cell RCC and papillary RCC specimens. A total of 3333 genes (16.2%) met condition (1), and a total of 4982 genes (24.3%) in the analysis of clear cell RCCs and a total of 5008 genes (24.4%) in the analysis of papillary RCCs met condition (2). Three genes satisfied condition (1) as well as condition (2) in both clear cell RCC and papillary RCC specimens and were considered to be chromophobe RCC-specific genes (Figure 1 and Table 2). The 3 genes were *BSND*, *ATP6V1G3*, and *FBN3*; as far as we know, all 3 of these genes have not been reported as genes specifically expressed in chromophobe RCC. These results indicate that our selection identified 3 novel candidate genes for differentiating between chromophobe RCC and other subtypes of RCC.

**TABLE 2:**
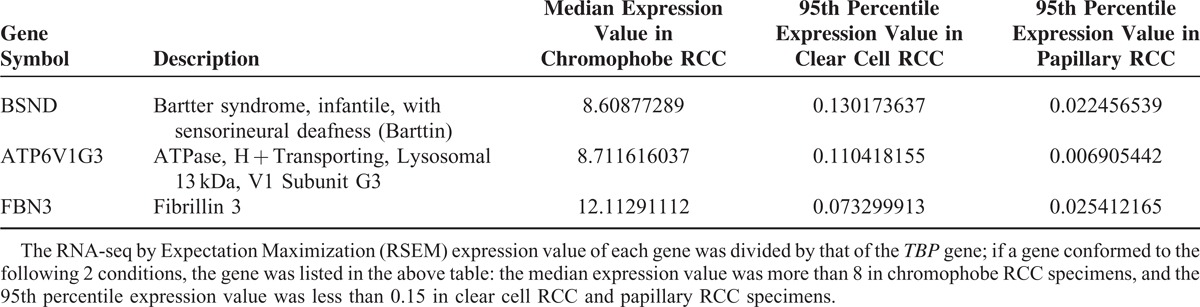
Genes Specifically Expressed in Chromophobe Renal Cell Carcinoma (RCC), Compared With Those Expressed in Clear Cell RCC and Papillary RCC

**FIGURE 2:**
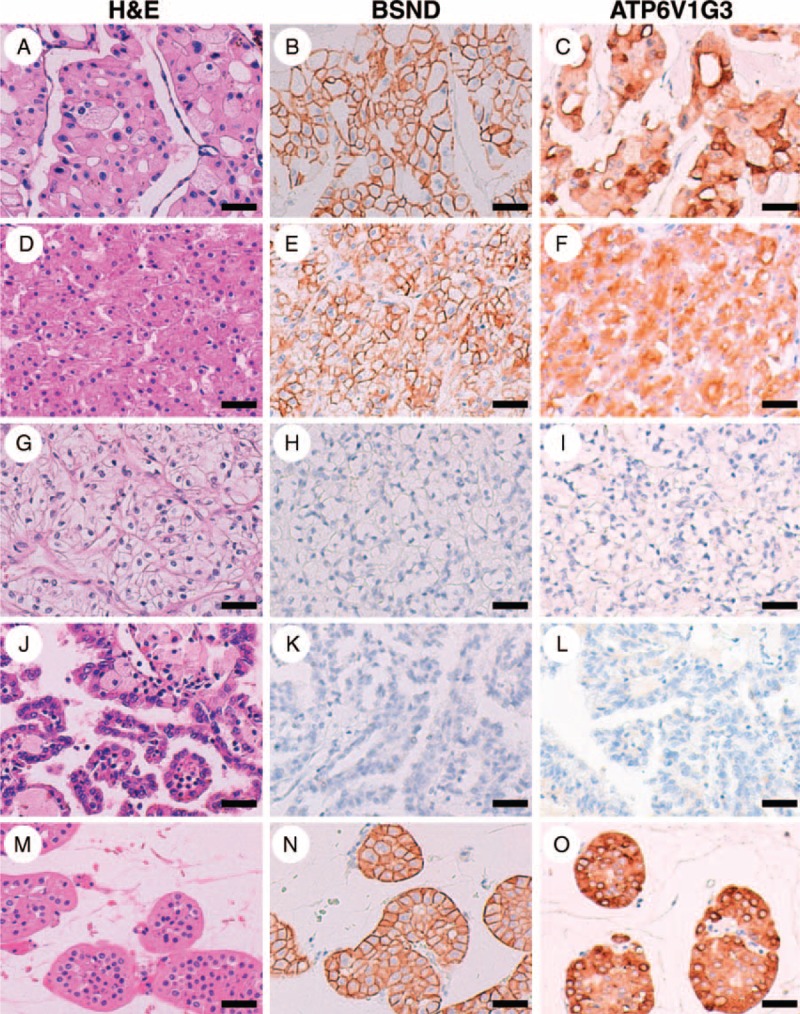
Typical immunohistochemical profile of BSND and ATP6V1G3 in renal epithelial tumors. Positive immunohistochemical staining for BSND and ATP6V1G3 was observed in a chromophobe RCC case (A–C) and an eosinophilic variant case of chromophobe RCC (D–F). Negative immunostaining was observed in a clear cell RCC case (G–I) and a papillary RCC case (J–L). Positive immunohistochemical staining was also observed in a renal oncocytoma case (M–O). Scale bar = 40 μm. RCC = renal cell carcinoma.

**FIGURE 3:**
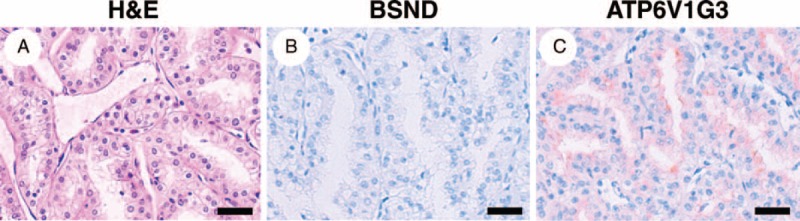
An example of a rare case of clear cell RCC showing weak ATP6V1G3 immunoreactivity. In this case, immunohistochemistry revealed negative BSND staining and weak ATP6V1G3 staining (A–C). Scale bar = 40 μm. RCC = renal cell carcinoma.

In general, the expression levels of mRNA and protein are not always correlated. So, we next examined whether the expression levels of the BSND, ATP6V1G3, and FBN3 proteins differed between chromophobe RCC and other RCC subtypes by performing an immunohistochemical analysis using a TMA technique. An immunohistochemical analysis using an antibody to FBN3, 1 of the 3 proteins, did not show any specific signal in RCCs or normal kidney tissues; therefore, we were not able to evaluate the protein expression status of FBN3. On the other hand, immunohistochemical analyses using antibodies to the other 2 proteins, BSND and ATP6V1G3, showed specific immunohistochemical signals in the membrane and cytoplasm of the tumor cells; BSND was preferentially expressed in the cell membrane in almost all the BSND-positive RCC cases, while ATP6V1G3 was expressed nearly equally in both the cell cytoplasm and membrane in most of the ATP6V1G3-positive RCC cases, but a subset of cases showed a predominance for expression in either the cytoplasm or the membrane (Figure 2A–L). Interestingly, strong diffuse positivity was observed in the immunohistochemical analyses for the BSND and ATP6V1G3 proteins in all the chromophobe RCC specimens (23/23 cases, 100%) but was not observed in the clear cell RCC specimens (0/153 cases, 0%) or the papillary RCC specimens (0/10 cases, 0%) (Figure 2A–L and Table 3). None of the clear cell or papillary RCC specimens showed even a weak positivity for BSND immunostaining; on the other hand, weak diffuse or partial positivity for ATP6V1G3 was detected in some clear cell RCC specimens (8/153 cases, 5.2%) and 1 papillary RCC specimen (1/10 cases, 10%) (Figure 3 and Table 3). Thus, when calculating the sensitivity and specificity using the immunohistochemical results based only on strong diffuse positivity, the sensitivity of BSND or ATP6V1G3 expression for the diagnosis of chromophobe RCC was 100%, and the specificity was 100%. If immunohistochemical results based on strong diffuse positivity and weak positivity were used, the specificity of ATP6V1G3 expression for the diagnosis of chromophobe RCC decreased slightly (94.5%). These results suggested that both BSND and ATP6V1G3 are excellent immunohistochemical markers for differentiating between chromophobe RCC and other RCC subtypes.

**TABLE 3:**
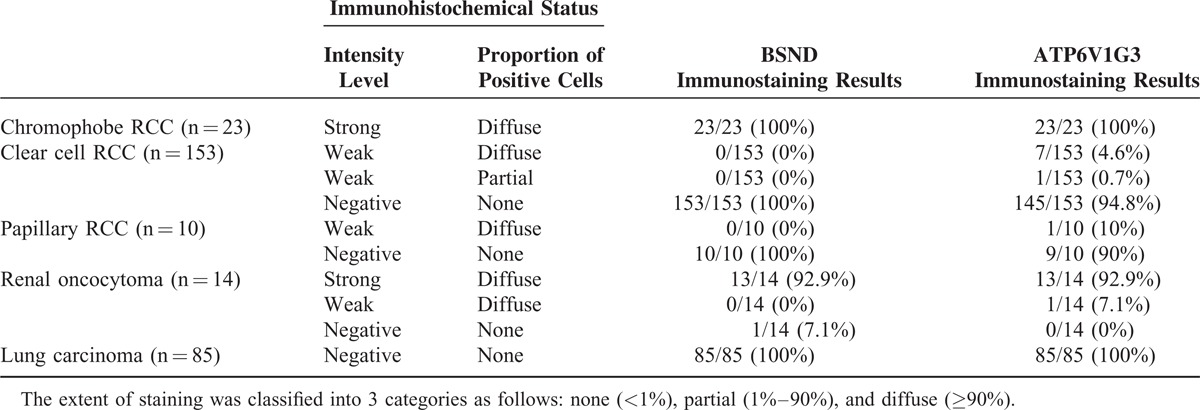
Immunohistochemical Results of BSND and ATP6V1G3 Proteins in Primary Renal Cell Tumors and Primary Lung Carcinomas

We next examined the expression status of BSND and ATP6V1G3 in renal oncocytoma, since this benign tumor often shares common morphological and immunophenotypic features with chromophobe RCC.^30,31^ Immunohistochemical analysis for the BSND and ATP6V1G3 proteins revealed strong diffuse positivity for both in most of the renal oncocytoma specimens (13/14 cases, 92.9%, for both proteins) (Figure 2M–O and Table 3), suggesting that BSND and ATP6V1G3 are immunohistochemical markers for renal oncocytoma as well as chromophobe RCC.

**FIGURE 4:**
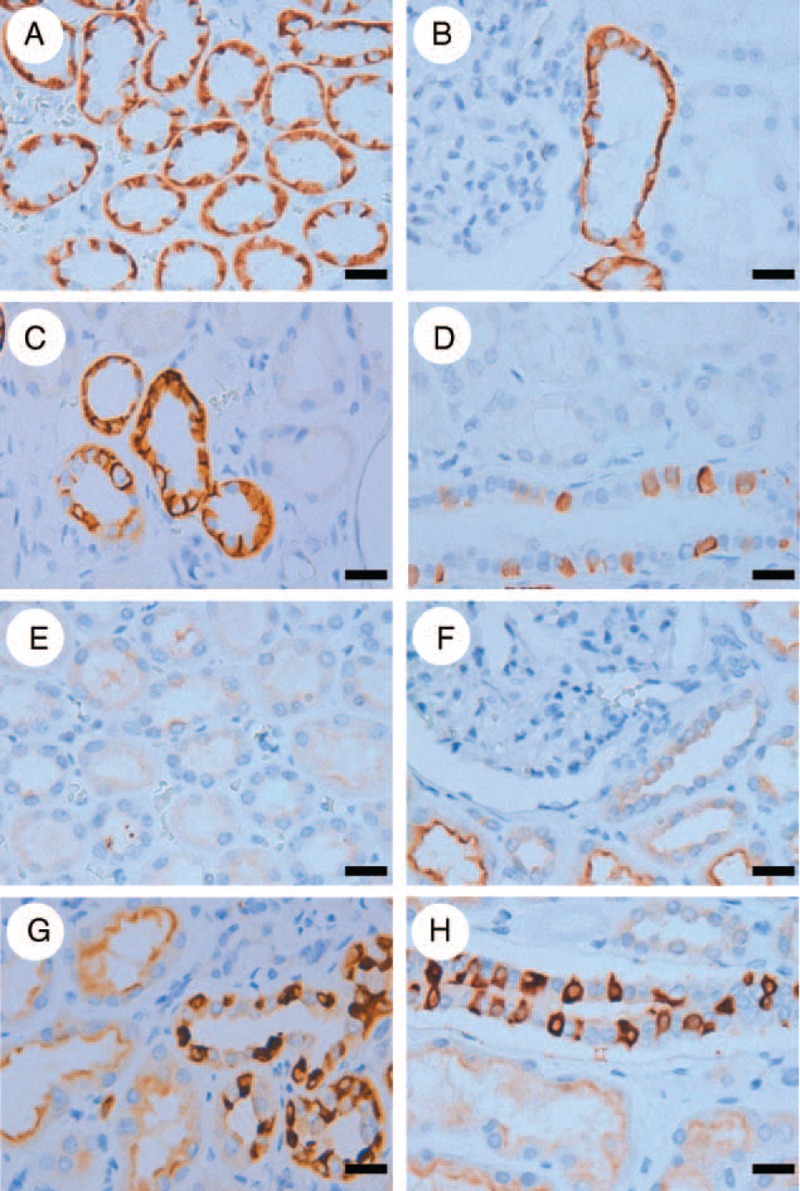
Typical immunohistochemical profile of BSND and ATP6V1G3 in noncancerous renal tissues. BSND was strongly expressed in the thin limb (A**)** and thick ascending limb (B**)** of the loop of Henle, the distal convoluted tubule (C**)**, and the collecting duct (D**)**, while ATP6V1G3 was strongly expressed in the distal convoluted tubule (G) and the collecting duct (H**)**, weakly in the proximal tubule and the thick ascending limb of the loop of Henle (F and G**)**, and very weakly in the thin limb of the loop of Henle (E**)**. Scale bar = 20 μm.

In the immunohistochemical analyses of renal tumors, we found that some components of normal kidney tissue were also immunoreactive for BSND and ATP6V1G3. BSND was strongly expressed in the thin limb and thick ascending limb of the loop of Henle, the distal convoluted tubule, and the collecting duct (Figure 4A–D). On the other hand, ATP6V1G3 was expressed at differential intensities in the nephrons: strong expression was observed in the distal convoluted tubule and collecting duct, while weak expression was observed in the proximal tubule and the thick ascending limb of the loop of Henle and very weak expression was observed in the thin limb of the loop of Henle (Figure 4E–H). BSND or ATP6V1G3 expression was not observed in the glomerular epithelium. These results suggested that BSND and ATP6V1G3 are variably expressed in normal kidney tissue, predominantly in the distal nephrons.

**FIGURE 5:**
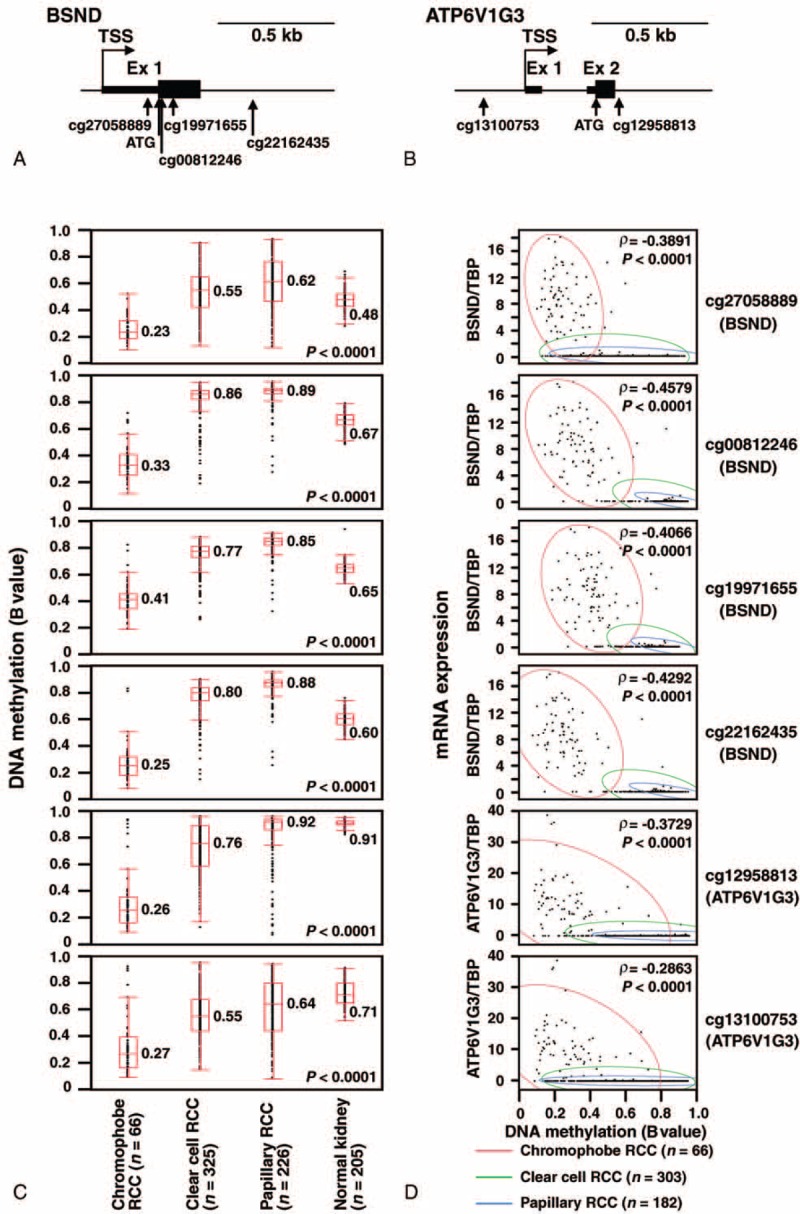
Decreased DNA methylation level and correlation of DNA methylation level with the expression levels of the *BSND* and *ATP6V1G3* genes in chromophobe RCC. (A, B) Map of the DNA methylation probes near the transcription start sites (TSSs) of the *BSND* (A) and *ATP6V1G3* (B) genes. The vertical arrows mark the position of the DNA methylation probes (CpG sites) or the translation initiation site (ATG). The thicker section in the exon region indicates the coding sequence. (C) Box plots of DNA methylation at CpG sites in the *BSND* and *ATP6V1G3* genes in 3 RCC subtypes and normal kidney. Statistically significant differences in the DNA methylation levels, which were shown as the β values, were detected among the groups (Kruskal-Wallis test). The median values are shown. (D) Dot plots of BSND or ATP6V1G3 expression and DNA methylation at the CpG sites of the *BSND* or *ATP6V1G3* gene. The expression levels are shown as the RSEM value of each gene, divided by that of the *TBP* gene. The DNA methylation level is shown as the β value. The Spearman rank correlation coefficient (ρ) and *P* values were provided. A bivariate normal ellipse (*P* = 0.95) was observed for each RCC subtype. RCC = renal cell carcinoma, RSEM = RNA-seq by Expectation Maximization.

We hypothesized that the differential expression levels of BSND and ATP6V1G3 among the 3 subtypes of RCC were partly attributable to DNA methylation. So, we tested this hypothesis by examining the DNA methylation level of the *BSND* and *ATP6V1G3* genes in 3 subtypes of RCC using data from the TCGA database. Four CpG sites (cg27058889, cg00812246, cg19971655, and cg22162435) near the transcription start site (TSS) of *BSND* and 2 sites (cg12958813 and cg13100753) near the *ATP6V1G3* TSS showed significantly lower DNA methylation levels (β values) in chromophobe RCC than in clear cell RCC and papillary RCC; these median β values of BSND or ATP6V1G3 in chromophobe RCC were lower than those in the other 2 RCCs by more than 0.25 (Figure 5A–C). Moreover, the β values in the above 6 CpG sites and the mRNA expression level in BSND or ATP6V1G3 were significantly correlated (Spearman ρ values −0.3891 to −0.4579 in BSND, and −0.2863 and −0.3729 in ATP6V1G3) (Figure 5D). These results suggested that DNA methylation is one of the mechanisms underlying the differential expression levels of BSND and ATP6V1G3 among the 3 subtypes of RCCs.

**FIGURE 6:**
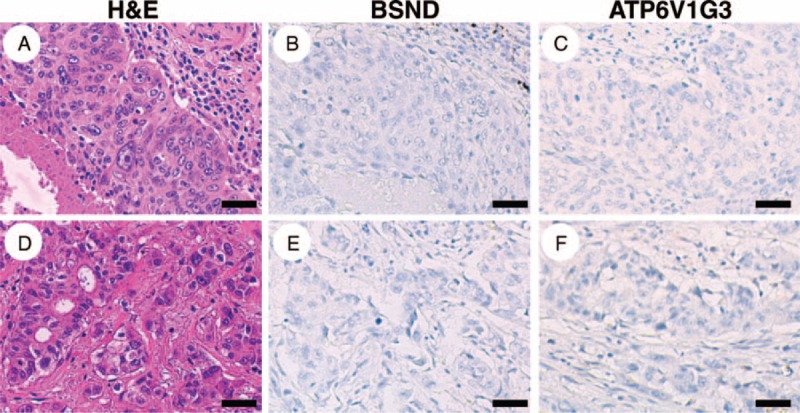
Typical immunohistochemical results of BSND and ATP6V1G3 in primary lung carcinoma. Both squamous cell carcinoma (A–C**)** and adenocarcinoma (D–F**)** cases were negative for BSND and ATP6V1G3 expression. Scale bar = 40 μm.

Although chromophobe RCC exhibits a better prognosis than conventional clear cell RCC,^2^ it can metastasize to distant organs including the lung.^6^ Thus, we examined the expression status of BSND and ATP6V1G3 proteins in lung carcinomas. The results showed that BSND and ATP6V1G3 protein was not expressed in a total of 85 lung carcinomas, composed of 44 cases of squamous cell carcinoma of the lung and 41 cases of adenocarcinoma of the lung (Figure 6 and Table 3). These results implied that BSND and ATP6V1G3 are excellent immunohistochemical markers for differentiating between chromophobe RCC that has metastasized to the lung and primary lung carcinoma.

Finally, to determine the expression levels of BSND and ATP6V1G3 in various types of carcinoma other than RCC and lung carcinoma, we examined the 95th percentile mRNA expression values of BSND and ATP6V1G3 in various types of carcinoma using data from the TCGA database. The 95th percentile expression values for bladder urothelial carcinoma, breast invasive carcinoma, cervical squamous cell carcinoma and endocervical adenocarcinoma, colon adenocarcinoma, head and neck squamous cell carcinoma, liver hepatocellular carcinoma, prostate adenocarcinoma, rectal adenocarcinoma, stomach adenocarcinoma, and thyroid carcinoma in addition to those of lung adenocarcinoma and lung squamous cell carcinoma were 1.9 × 10^−3^ to 3.4 × 10^−2^ for the *BSND* gene and 0 to 1.6 × 10^−2^ for the *ATP6V1G3* gene; these values for the 12 above-mentioned types of carcinoma were much lower than those for chromophobe RCC (14.7 for BSND and 26.7 for ATP6V1G3) (Table 4). These results suggested that the expression levels of BSND and ATP6V1G3 were extremely low in various types of carcinoma.

**TABLE 4:**
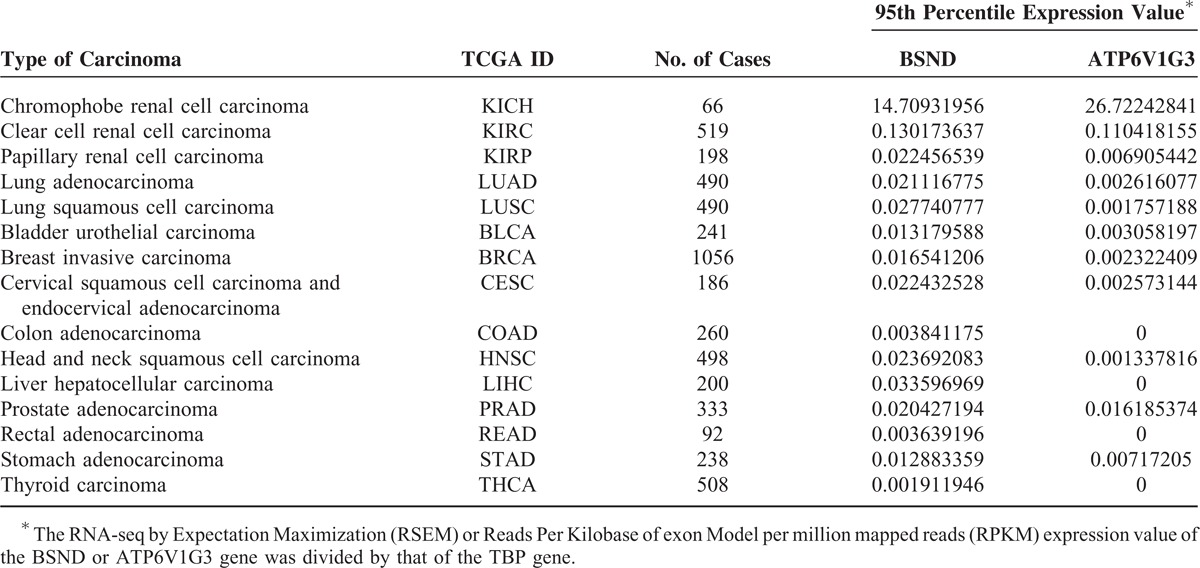
Ninety Fifth Percentile mRNA Expression Value in Various Types of Carcinoma Using Data From the TCGA Database

## DISCUSSION

In this study, 3 genes including *BSND* and *ATP6V1G3* were identified as being specifically expressed in chromophobe RCC at the mRNA level using RNA-seq expression data from the TCGA database. Further immunohistochemical analysis of the protein expression levels of these 3 genes in specimens from our RCC series revealed that BSND and ATP6V1G3 were strongly and diffusely expressed in all the chromophobe RCC specimens (100%) but not in the clear cell or papillary RCC specimens (0% each). Although weak positivity for ATP6V1G3 was detected in a subset of clear cell RCC (5.2%) and papillary RCC (10%), none of the clear cell or papillary RCC specimens showed even a weak positive signal for BSND. DNA methylation was suggested to be one of the mechanisms underlying the differential expression pattern seen among the 3 subtypes of RCC. Most renal oncocytoma specimens (92.9%) also showed BSND and ATP6V1G3 protein expression. In normal kidney, BSND and ATP6V1G3 protein was expressed mainly in the distal nephron. Regarding the expression levels of BSND and ATP6V1G3 in carcinomas other than RCC, lung carcinomas were negative (0%) for these protein expressions when examined using immunohistochemical analyses, and the TCGA data showed that the mRNA expression levels of both genes were extremely low in 12 types of carcinoma, including lung carcinoma. These results suggest that BSND and ATP6V1G3 might be useful immunohistochemical markers for the differential diagnosis of chromophobe RCC. The current study is the first to report the immunohistochemical status of BSND and ATP6V1G3 in chromophobe RCC, and we consider that both immunomarkers might be applicable for routine pathology laboratory studies.

In the current study, the sensitivity of BSND or ATP6V1G3 expression for the diagnosis of chromophobe RCC was 100%, and the specificity was 100%, when calculated based only on strong diffuse positivity. These values for BSND and ATP6V1G3 are superior or equal to those of any other immunohistochemical marker that has been used previously for the differential diagnosis of chromophobe RCC.^10–21,32^ Among the numerous markers identified for such differential diagnosis, CK7 and AMACR are currently widely accepted. When compared among clear cell, papillary, and chromophobe RCCs, the reported sensitivity of CK7 expression for the diagnosis of chromophobe RCC is in the range of 65.9% to 100% and the specificity is in the range of 63.1% to 88.9%;^10,13,33–36^ the reported sensitivity of AMACR expression for the diagnosis of papillary RCC is 100% and the specificity is in the range of 81.4% to 86.7%.^37–39^ Thus, both BSND and ATP6V1G3 immunohistochemistry may be used for routine pathological diagnosis, just like CK7 and AMACR. In the comparison of the diagnostic usefulness between BSND and ATP6V1G3, a subset of clear cell RCC and a subset of papillary RCC cases showed weak ATP6V1G3 expression in the tumor cells; therefore, BSND immunohistochemistry may be more reliable than ATP6V1G3 immunohistochemistry from the perspective of diagnostic utility. We are planning to carry out BSND and ATP6V1G3 immunohistochemistry in a larger number of cases and to determine whether the immunohistochemical statuses of these markers might be associated with the clinicopathological factors, including survival, in the future.

In our analysis, renal oncocytoma was also found to be positive at a high frequency (92.9%) for BSND and ATP6V1G3 immunostaining. Since this benign tumor often shares common morphological features with chromophobe RCC, the differential diagnosis between the 2 conditions is important. However, based on our results, BSND or ATP6V1G3 immunohistochemistry is not useful for differentiating between chromophobe RCC and renal oncocytoma.

BSND encodes the β-subunit of ClC-K chloride channels, which play an important role in chloride transport in the kidney and inner ear.^40^ Germline mutations of the *BSND* gene cause Bartter syndrome type IV, which is an autosomal recessive disease characterized by salt loss, hypokalemia, metabolic alkalosis, and sensorineural deafness.^41^ At present, several research papers examining germline mutations of the *BSND* gene in the Bartter syndrome family have been reported;^42^ however, the expression of BSND protein in RCC has not been previously reported. ATP6V1G3, another immunohistochemical marker identified in this study, is a subunit of vacuolar-H^+^ ATPase that couples ATP hydrolysis to proton pumping across membranes.^43,44^ In the kidney, vacuolar-H^+^ ATPase has an important role in the regulation of acid/base balance.^44,45^ Clinically, a reduction in the mRNA expression of ATP6V1G3 in clear cell RCC has been previously reported;^46^ however, its expression status in chromophobe RCC has not been previously reported. Thus, our paper is the first to report that these 2 proteins that are physiologically involved in membrane transport, BSND and ATP6V1G3, are differentially expressed among the 3 main subtypes of RCCs. As another aspect, our findings that both proteins were expressed chiefly in the distal nephron, including the collecting duct, of normal kidney tissue and specifically in chromophobe RCC among RCCs may strengthen the previously proposed idea that chromophobe RCC is derived from the distal nephron, specifically the collecting duct.^47^

Microarray analyses have been used in some previous reports to identify novel diagnostic immunohistochemical markers for chromophobe RCC.^11,14,15,48^ However, BSND and ATP6V1G3 have not been detected as genes specifically expressed in chromophobe RCC, compared with those expressed in clear cell RCC. Several reasons for these differences may exist: a comparison of chromophobe RCC versus other RCC subtypes, but not chromophobe RCC versus normal kidney, was performed in the present study; the RNA-seq method was applied in the present study, whereas the hybridization method was used in previous studies; and the number of examined cases was larger in the present study than in the previous studies. Considering the successful identification of immunomarkers in our study, different comparisons of the RNA-seq expression data from the TCGA database, such as a search for genes showing high expression levels in clear cell RCC and low expression levels in chromophobe and papillary RCCs, could lead to the identification of further novel immunomarkers that are useful for the pathological diagnosis of RCC.

In this study, an examination of mRNA expression and DNA methylation data from the TCGA database suggested that DNA methylation might be one of the factors causing the difference in BSND and ATP6V1G3 expressions among the RCC subtypes. Davis et al^25^ recently reported that the DNA methylation profile inversely correlated with mRNA expression is globally distinct between chromophobe RCC and clear cell RCC. We suspect that both BSND and ATP6V1G3 are members of the group of genes whose expressions are differentially influenced by the DNA methylation status between chromophobe RCC and other RCC subtypes, such as clear cell and papillary RCCs. Since our examination of the DNA methylation status of *BSND* and *ATP6V1G3* in RCC was an in silico analysis, future alternative experimental analyses, such as methylation-specific polymerase chain reaction, towards CpG sites near the TSS of *BSND* and ATP6V1G3 would further endorse our suggestion on the relationship between DNA methylation and the expressions of these genes.

An examination of the mRNA expression data from the TCGA database also revealed that the mRNA expression levels of BSND and ATP6V1G3 were extremely low in various human carcinomas in this study. In practice, the expression of neither of these proteins was observed in lung carcinoma in our immunohistochemical analysis. Since chromophobe RCC can metastasize to various distant sites,^6^ this information could be helpful for discerning whether chromophobe RCC has metastasized to an organ or a primary carcinoma of that organ is present. For the better application of BSND and ATP6V1G3 immunohistochemistry in practical pathological diagnosis, whether BSND or ATP6V1G3 positivity is observed in any tumor other than the 12 types of carcinoma examined in the current study is now being investigated in our laboratory.

In conclusion, we have identified, for the first time, the utility of BSND and ATP6V1G3 as immunohistochemical markers for the differential diagnosis of chromophobe RCC from other RCC subtypes, such as clear cell and papillary RCCs. Additionally, our results suggest that both immunomarkers may be useful for identifying the metastasis of chromophobe RCC to distant organ sites.
